# Association between the ratio of serum eicosapentaenoic acid to arachidonic acid and risk of coronary artery disease in young Chinese patients

**DOI:** 10.3389/fnut.2022.1019058

**Published:** 2022-11-03

**Authors:** Xiong Liu, Lichang Sun, Weixing Wen, Min Qiu, Jianjing Luo, Weiwen Li, Shali Hao, Mingli He, Jiandi Wu, Yunzhao Hu, Yuli Huang

**Affiliations:** ^1^Department of Cardiology, Shunde Hospital, Southern Medical University, Foshan, China; ^2^Department of Internal Medicine, Zhaoqing Medical College, Zhaoqing, China; ^3^Department of Cardiology, Affiliated Foshan Hospital, Southern Medical University, Foshan, China; ^4^Guangdong Provincial Key Laboratory of Cardiac Function and Microcirculation Research, Guangzhou, China

**Keywords:** eicosapentaenoic acid, docosahexaenoic acid, arachidonic acid, risk factors, coronary artery disease, young

## Abstract

**Objective:**

Long-chain (LC) omega-3 PUFAs, including eicosapentaenoic acid (EPA) and docosahexaenoic acid (DHA), may play an anti-inflammatory effect and decrease the risk of coronary artery disease (CAD). In contrast, omega-6 PUFA, mainly arachidonic acid (AA), has pro-inflammatory and pro-aggregatory effects, which may increase the risk of CAD. This study evaluated the associations between EPA, DHA, AA, and their ratios (EPA/AA and DHA/AA) with the risk of CAD in young Chinese patients.

**Methods:**

A total of 182 young patients with CAD and 143 age-matched controls were included. Traditional cardiovascular risk factors were recorded. Serum EPA, DHA and AA were measured by ultra-performance liquid chromatography-mass spectrometry.

**Results:**

The level of AA was significantly higher, while the level of EPA was lower in the CAD group than that in the control group. There was no significant difference in DHA level in the two groups. Both the ratios of EPA/AA and DHA/AA were lower in the CAD group than that in the control. Multivariate logistic regression analysis showed that higher serum AA level was associated with the increased risk of CAD, while EPA was a protective factor for CAD. There was no significant association between DHA level and the risk of CAD. Although both higher ratios of EPA/AA [per tertile increment, adjusted odds ratios (ORs) (OR) 0.356, 95% confidence intervals (CI) 0.247–0.513] and DHA/AA (adjusted OR = 0.465, 95%CI = 0.332–0.653) were associated with a lower risk of CAD in young patients. Receiver operating characteristic (ROC) curve analysis showed that compared with AA, the diagnostic value was increased in EPA/AA, but not in DHA/AA.

**Conclusion:**

EPA, but not DHA may play a protective role in CAD, while AA may be associated with the increased risk of CAD in young Chinese patients. The ratio of EPA/AA can increase the predictive value for diagnosing CAD than EPA or AA alone.

## Introduction

Cardiovascular disease (CVD) had become a major health burden and cause of mortality worldwide (1). Although generally prevalent in old age individuals, the prevalence of CVD, especially coronary artery disease (CAD) in younger people had been increasing during the past decades (2, 3). The clinical characteristics and risk factors of CAD are quite different between young and older patients. Conventional cardiovascular risk factors [e.g., hypertension, diabetes mellitus (DM), and dyslipidemia] are less prevalent in young CAD patients (4). A previous study showed that only 36% of young patients attacked with myocardial infarction had no or only one conventional cardiovascular risk factor, thus would be mistakenly classified as low risk if only based on the traditional risk scoring system (5). Therefore, detection and proper management of novel risk factors for CAD in young patients are of vital importance to decrease the global burden of CVD.

Polyunsaturated fatty acids (PUFA) play an important role in cardiovascular health. Long-chain (LC) omega-3 PUFAs, including eicosapentaenoic acid (EPA) and docosahexaenoic acid (DHA), most commonly found in seafood or fish oil, may play an anti-inflammatory effect and decrease the risk of CAD. In contrast with omega-3 PUFA, omega-6 PUFA, mainly arachidonic acid (AA), has pro-inflammatory and pro-aggregatory effects in the human body, which may increase the risk of CAD. Therefore, the ratio of omega-3/omega-6 PUFA may reflect the balance of anti-inflammatory and pro-inflammatory fatty acids in circulation, which may significantly relate to cardiovascular risk. Observational studies from Japan, a country with a large amount of seafood intake, showed that a low EPA/AA ratio is associated with an increased risk of CVD, including CAD, stroke, and PAD (6–9), especially in young individuals. However, whether such association was true in the Chinese population was still unclear. Furthermore, EPA and DHA may play different physical effects, and whether they are similar in the risk of CVD was also controversal (7, 10–12).

Therefore, we evaluated the relation between EPA, DHA, and AA with the risk of CAD in young Chinese patients. We also explored whether the combination of these free fatty acids, calculated as the ratio of EPA/AA and DHA/AA, can increase the predictive value for diagnosing CAD.

## Materials and methods

### Participants

This study was conducted complying with the Declaration of Helsinki and was approved by the Ethics Committee of Shunde Hospital, Southern Medical University, China (NO: KY20191103). Written informed consent was obtained from all participants. All the participants were recruited from the participated hospitals. Young patients with CAD were defined as those presenting with initial CAD symptoms at ≤ 55 or ≤ 65 years of age in men or women, respectively. CAD was diagnosed as ≥ 50% stenosis of the lumen diameter in at least one major coronary artery (including the left main coronary artery, left anterior descending branch, left circumflex branch and right coronary artery. Two independent interventional cardiologists evaluated coronary artery stenosis), which was quantified by coronary angiography (CAG). CAG was performed using the Judkins technique through the radial artery, if failed, the femoral artery access was chosen as an alternative. The results of CAG were assessed by two independent interventional cardiologists, and further evaluated by one radiologist from the participated hospitals. Hospitalized age-matched individuals without a diagnosis of CAD in the same period were screened and included as the controls.

Patients were excluded if suspected acute myocarditis or stress cardiomyopathy; uncontrolled infectious disease, autoimmune disease, end-stage renal disease, acute hepatitis, psychiatric disorders, or malignancy; or received fish oil or polyunsaturated fatty acid supplement during the past 3 months.

### Laboratory detection and definition of covariates for coronary artery disease

Levels of hemoglobin (HgB), platelets, fasting blood glucose (FBG), glycated hemoglobin (HbA1c), alanine aminotransferase (ALT), aspartate aminotransferase (AST), total cholesterol (TC), high-density lipoprotein cholesterol (HDL-C), triglyceride (TG), level of low-density lipoprotein cholesterol (LDL-C), and serum creatinine (Scr) were measured in the laboratory departments of the participated hospitals, and extracted from the medical records.

Conventional risk factors for CAD included as covariates in our study were as follows: (1) Family history of premature CAD was defined as a diagnosis of CAD in a first-degree male relative aged < 55 years or female relative aged < 65 years. (2) Hypertension was defined according to the current Chinese guidelines for the management of hypertension (13), including those with a systolic blood pressure ≥ 140 mmHg and/or diastolic blood pressure ≥ 90 mmHg, or who had received antihypertensive treatment. (3) Type 2 DM was defined as an FBG of ≥ 7.0 mmol/L, or HbA1c of ≥ 6.5%, or treatment with hypoglycemic medication (14). (4) Dyslipidemia was defined as TC of ≥ 5.18 mmol/L, LDL-C of ≥ 3.37 mmol/L, HDL-C of < 1.04 mmol/L, and/or TG of ≥ 1.7 mmol/L or a history of anti-dyslipidemia treatment (15). (5) For cigarette smoking, participants were classified as smokers if they smoked regularly during the past year. Those who had never smoked or stopped smoking for more than 1 year were classified as non-smokers.

### Detections of eicosapentaenoic acid, docosahexaenoic acid, and arachidonic acid

Venous blood samples were collected after at least 8 h of fasting and stored at −80°C for future measurements of free fatty acid. Detections of DHA, EPA, and AA were performed in a commercial company (BiotechPack ANALYTICAL, Beijing, China) using ultra-performance liquid chromatography-mass spectrometry methods according to previous reports (16, 17). In brief, the stored serum samples were thawed at 4°C, and 50 mg of the samples were homogenized with 100 μL distilled water. Added with 0.5 ml of methanol, samples were extracted by vortexing for 30 min. After centrifuging at 14,000 rpm at 4°C for 5 min, the supernatant was added with 5 μL of the inter-standard solution (FA19:0 25 μg/ml, diluted with methanol), then vortexed for 10 s, stored in a 2 ml injection vial for the test.

After that, the UPLC analysis was performed using a Waters ACQUITY I-class LC system (Waters, Milford, MA, USA). Chromatographic separation was conducted on a Waters ACQUITY UPLC BEH C18 column (1.7 μm particle size, 2.1 mm × 100 mm), maintained at 55°C. The mobile phase consisted of solvent A (Acetonitrile: water, 1:10, 1 mmol/L ammonium acetate) and solvent B (Isopropanol: Acetonitrile, 1:1). Gradient elution was carried out at a flow rate of 0.30 ml/min, with the injection volume of 1 μL. Mass spectrometry was performed using a Xevo TQ-S micro spectrometer (Waters, Milford, MA, USA). The following negative ion ESI parameters were used: turbo spray temperature 150°C, spray voltage –2.5 kV, cone voltage 21 V, desolvation temperature 500°C, and desolvation gas flow 1,000 L/h.

The system was controlled by the Masslynx Analysis software (version 4.1, SCIEX, Boston, MA, USA). The Skyline software (MacCoss, WA, USA) was used to analyze the raw data.

### Statistical analysis

Categorical variables were presented as numbers and percentages. Continuous variables were presented as mean ± standard deviation (SD) or median and interquartile range (IQR). Baseline characteristics of CAD patients and controls were compared by the Wilcoxon rank-sum test for non-normally distributed continuous variables, two-tailed *t*-test for normally distributed continuous variables, and the chi-square test with Yates’ correction for continuity or Fisher’s exact test for categorical variables, as appropriate.

Correlations between covariates and AA, DHA and EPA were evaluated by the Pearson product-moment correlation coefficient (*r*). In this analysis, variables with non-Gaussian distribution were logarithmically transformed. To evaluate the association between the serum free fatty acids and the risk of CAD, EPA, DHA, AA, EPA/AA, and DHA/AA were divided into tertiles according to their levels, respectively. Multivariate logistic regression analysis was used to evaluate the associated factors for CAD, with adjustment of age, sex, smoking, hypertension, diabetes mellitus, TC, TG, HDL-C, and LDL-C using an enter method. The adjusted odds ratios (ORs) and 95% confidence intervals (95%CIs) were calculated.

A receiver operating characteristic (ROC) curve was performed, and the area under the curve (AUC) was calculated to evaluate the diagnostic value of EPA, DHA, AA, EPA/AA, and DHA/AA for CAD in young patients. Pairwise comparisons of ROC curves were performed according to the method proposed by Hanley and Hajian-Tilaki (18).

All the statistical analysis was performed using SPSS Statistics for Windows (Version 23.0, IBM Corp., Armonk, NY, USA) and MedCalc (Version 20.0, MedCalc Software Ltd., Belgium). All *P*-values were two-sided, and a *P* < 0.05 was considered statistically significant.

## Results

### Clinical characteristics of the patients

In this case-control study, we included 182 young patients with CAD (83.0% male) and 143 age-matched controls (44.1% male) for analysis, according to the predefined inclusion criteria. The baseline demographic and clinical characteristics of all the participants are shown in Table 1. The median age of all the participants was 49.0 (IQR 45.0, 53.0) years old, similar in the CAD group (median 49.0, IQR 45.0, 53.0) and the control group (median 50.0, IQR 45.0, 54.0) (*P* = 0.456). There was a higher proportion of male sex, current smokers, and DM in patients with CAD compared with the controls. Furthermore, the levels of FBG, HbA1c, TG, ALT, and AST were higher, while the level of HDL-C was lower in the CAD patients than those in the control group (all *P* < 0.05). There were no significant differences in other traditional risk factors of CVD between the two groups.

**TABLE 1:** Demographic and clinical characteristics of CAD patients and controls.

	All participants (*n* = 325)	CAD group (*n* = 182)	Control group (*n* = 143)	*P-*value
Age (years)	49.0 (45.0, 53.0)	49.0 (45.0, 53.0)	50.0 (45.0, 54.0)	0.456
Men [n(%)]	214 (65.8%)	151 (83.0%)	63 (44.1%)	< 0.001
CAD family history [n(%)]	20 (6.2%)	9 (4.9%)	11 (7.7%)	0.429
Current smokers [n(%)]	129 (39.7%)	90 (49.5%)	39 (27.3%)	0.0001
HR (beats/minute)	76.0 (67.0, 86.0)	76.0 (66.0, 86.0)	77.0 (70.0, 86.0)	0.241
Hypertension [n(%)]	169 (52.0%)	97 (53.3%)	72 (50.3%)	0.677
SBP (mm Hg)	127.0 (115.0, 144.0)	126.0 (114.0, 140.0)	131.0 (116.25, 147.0)	0.106
DBP (mm Hg)	82.0 (73.75, 91.0)	82.0 (72.0, 91.0)	81.0 (75.0, 91.0)	0.840
DM [n(%)]	90 (27.7%)	59 (32.4%)	31 (21.7%)	0.043
FBG (mmol/L)	5.80 (5.10, 7.44)	5.93 (5.15, 8.12)	5.69 (5.05, 6.79)	0.024
HbA1c (%)	5.8 (5.4, 6.21)	5.8 (5.5, 6.4)	5.7 (5.4, 6.1)	0.030
HgB (g/L)	138.0 (125.0, 147.0)	139.0 (127.0, 147.0)	133 (121.0, 147.0)	0.043
TC (mmol/L)	4.37 (3.71, 5.27)	4.24 (3.60, 5.23)	4.51 (3.96, 5.28)	0.163
LDL-C (mmol/L)	2.58 (2.06, 3.11)	2.58 (2.04, 3.28)	2.56 (2.09, 2.93)	0.312
HDL-C (mmol/L)	1.05 (0.87, 1.24)	0.98 (0.85, 1.19)	1.13 (0.95, 1.30)	0.001
TG (mmol/L)	1.47 (1.07, 2.08)	1.59 (1.17, 2.34)	1.35 (0.98, 1.88)	< 0.001
PLT (×10^9/L)	237.17 ± 66.0	243.18 ± 62.90	229.53 ± 69.23	0.064
ALT (U/L)	24.0 (18.0, 46.72)	28.0 (20.0, 94.0)	22.0 (16.0, 29.0)	< 0.001
AST (U/L)	28.0 (19.0, 46.0)	31.84 (21.0, 56.37)	23.0 (17.0, 34.95)	< 0.001
Scr (μmol/L)	75.5 (65.80, 89.0)	76.31 (68.0, 88.4)	72.58 (61.25, 90.05)	0.068
AA (μmol/L)	6.38 (4.31, 8.89)	7.26 (4.70, 10.23)	5.91 (3.90, 7.23)	< 0.001
EPA (μmol/L)	0.58 (0.38, 0.77)	0.53 (0.37, 0.74)	0.63 (0.44, 0.80)	0.017
DHA (μmol/L)	2.42 (1.58, 3.47)	2.44 (1.36, 3.60)	2.37 (1.78, 3.34)	0.755
EPA/AA	0.09 (0.07, 0.12)	0.08 (0.06, 0.10)	0.11 (0.08, 0.15)	< 0.001
DHA/AA	0.38 (0.28, 0.51)	0.35 (0.25, 0.44)	0.46 (0.33, 0.58)	< 0.001

Data are presented as percentages, mean and SD, median and interquartile range. AA, arachidonic acid; ALT, alanine aminotransferase; AST, aspartate aminotransferase; CAD, coronary artery disease; DBP, diastolic blood pressure; DM, diabetes mellitus; DHA, docosahexaenoic acid; DHA/AA, ratio of DHA and AA; EPA, eicosapentaenoic acid; EPA/AA, ratio of EPA and AA; FBG, fasting blood glucose; HDL-C, high density lipoprotein-cholesterol; HgB, hemoglobin; HR, heart rate; LDL-C, low density lipoprotein-cholesterol; PLT, platelets; SBP, systolic blood pressure; Scr, serum creatinine; TC, total cholesterol; TG, triglyceride.

Ultra-performance liquid chromatography-mass spectrometry based analysis showed that the level of AA was significantly higher (7.26 vs. 5.94 μmol/L, *P* < 0.001), while the level of EPA was lower (0.53 vs. 0.63 μmol/L, *P* = 0.017) in the CAD group than that in the control group. There was no significant difference in DHA level in the two groups. Both the ratios of EPA/AA (0.08 vs. 0.11, *P* < 0.001) and DHA/AA (0.25 vs. 0.46, *P* < 0.001) were significantly lower in the CAD group than that in the control (Table 1).

### Correlation between serum arachidonic acid level and other baseline variables

The correlations between serum AA level and other baseline clinical variables were presented in Table 2. We only found that the level of AA was negatively correlated with female (*r* = –0.13, *P* = 0.019), but not with other covariates, including SBP, DBP, FBG, HbA1C, TC, HDL-C, TG or Scr (all *P* > 0.05). However, there was a significant positive correlation between AA level and EPA (*r* = 0.534, *P* < 0.001), as well as DHA level (*r* = 0.746, *P* < 0.001).

**TABLE 2:** Correlation of arachidonic acid and other baseline variables.

Variables	*R*-value	*P*-value
Age	0.015	0.978
Female	–0.130	0.019
Smoking	0.106	0.056
SBP	–0.046	0.410
DBP	0.021	0.708
FBG	0.072	0.197
HbA1C	0.062	0.266
TC	–0.088	0.115
LDL-C	–0.042	0.450
HDL-C	0.017	0.757
TG	–0.093	0.093
Scr	–0.020	0.717
EPA	0.534	<0.001
DHA	0.746	<0.001

Age, AA, SBP, DBP, FBG, HbA1C, TC, HDL-C, LDL-C, TG, Scr, EPA, and DHA were skewed variables and logarithmically transformed. DBP, diastolic blood pressure; DHA, docosahexaenoic acid; DEPA, eicosapentaenoic acid; FBG, fasting blood glucose; HDL-C, high density lipoprotein-cholesterol; LDL-C, low density lipoprotein-cholesterol; PLT, platelets; SBP, systolic blood pressure; Scr, serum creatinine; TC, total cholesterol; TG, triglyceride.

### Association of serum eicosapentaenoic acid, docosahexaenoic acid, arachidonic acid, and their ratios with coronary artery disease in young patients

Multivariate logistic regression analysis showed that after adjustment of age, sex, smoking, hypertension, diabetes mellitus, TC, TG, HDL-C, and LDL-C, higher serum AA level (per tertile increment, adjusted OR = 1.593, 95%CI = 1.149–2.209) was associated with the increased risk of CAD in young patients, while higher level EPA was a protective factor for CAD (adjusted OR = 0.675, 95%CI = 0.486–0.937). There was no significant association between DHA level and risk of CAD (adjusted OR = 0.873, 95%CI = 0.636–1.198). Interestingly, both higher levels of EPA/AA (per tertile increment, adjusted OR = 0.356, 95%CI = 0.247–0.513) and DHA/AA (adjusted OR = 0.465, 95%CI = 0.332–0.653) were associated with a lower risk of CAD in young patients (Table 3).

**TABLE 3:** Association of serum EPA, DHA, AA and their ratios with CAD in young patients by multivariate logistic regression analysis.

Risk factors	Adjusted OR	95% CI	*P*-value
AA (per tertile increment)	1.593	1.149–2.209	0.005
EPA (per tertile increment)	0.675	0.486–0.937	0.019
DHA (per tertile increment)	0.873	0.636–1.198	0.40
EPA/AA (per tertile increment)	0.356	0.247–0.513	<0.0001
DHA/AA (per tertile increment)	0.465	0.332–0.653	<0.0001

Data were adjusted by age, sex, smoking, hypertension, diabetes mellitus, TC, TG, HDL-C, and LDL-C. AA, arachidonic acid; CAD, coronary artery disease; DHA, docosahexaenoic acid; DHA/AA, ratio of DHA and AA; EPA, eicosapentaenoic acid; EPA/AA, ratio of EPA and AA; HDL-C, high density lipoprotein-cholesterol; LDL-C, low density lipoprotein-cholesterol; TC, total cholesterol; TG, triglyceride.

**FIGURE 1:**
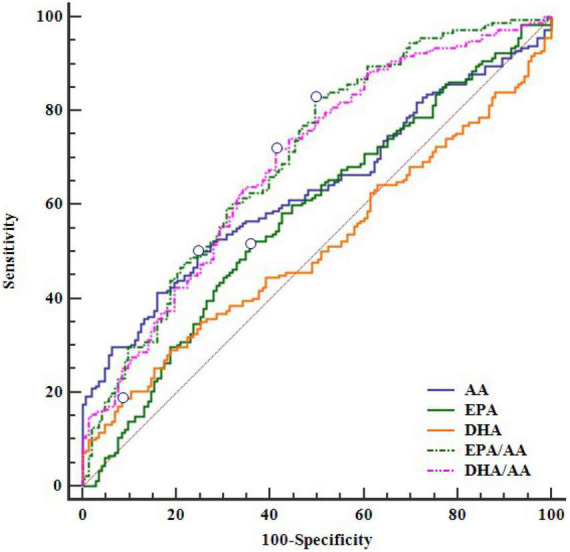
The ROC curve of EPA, DHA, AA and their ratios for CAD. AA, arachidonic acid; CAD, coronary artery disease; DHA, docosahexaenoic acid; DHA/AA, ratio of DHA and AA; EPA, eicosapentaenoic acid; EPA/AA, ratio of EPA and AA; ROC, receiver operating characteristic.

ROC analysis showed the diagnostic value of serum EPA, DHA, AA and their ratios for CAD in young patients (Figure 1). In decreasing order of AUC, EPA/AA (AUC = 0.700, *P* < 0.0001), DHA/AA (AUC = 0.690, *P* < 0.0001), AA (AUC = 0.630, *P* < 0.0001), and EPA (AUC = 0.577, *P* = 0.017), but not DHA (AUC = 0.510, *P* = 0.753) showed a significant effective value for predicting CAD in young patients (Table 4). Furthermore, pairwise comparisons of ROC curves showed that the EPA/AA ratio was more predictive than EPA [difference between areas (DBA) = 0.123, *P* = 0.0001] or AA (DBA = 0.070, *P* = 0.031] alone, while DHA/AA ratio was similar with the AA (DBA = 0.06, *P* = 0.137) level to predict the risk of CAD in young patients (Table 5).

**TABLE 4:** ROC curves of serum EPA, DHA, AA and their ratios for diagnosis of CAD in young patients.

Variable	AUC	95% CI	*P*-value
EPA/AA	0.700	0.647–0.749	< 0.0001
DHA/AA	0.690	0.637–0.740	< 0.0001
AA	0.630	0.575–0.683	< 0.0001
EPA	0.577	0.521–0.631	0.017
DHA	0.510	0.454–0.566	0.753

AA, arachidonic acid; CAD, coronary artery disease; DHA, docosahexaenoic acid; DHA/AA, ratio of DHA and AA; EPA, eicosapentaenoic acid; EPA/AA, ratio of EPA and AA; ROC, receiver operating characteristic.

**TABLE 5:** Pairwise comparisons of ROC curves.

Comparison	Difference between areas	95% CI	*P*-value
AA vs. EPA	0.053	–0.055–0.161	0.335
AA vs. DHA	0.120	0.005–0.235	0.041
EPA vs. DHA	0.067	0.018–0.115	0.007
EPA/AA vs. AA	0.070	0.006–0.134	0.031
EPA/AA vs. EPA	0.123	0.060–0.187	0.0001
DHA/AA vs. AA	0.060	–0.019–0.139	0.137
DHA/AA vs. DHA	0.180	0.120–0.239	< 0.0001
EPA/AA vs. DHA/AA	0.010	–0.043 to 0.064	0.709
DHA/AA vs. EPA	0.113	0.046 to 0.181	0.001
EPA/AA vs. DHA	0.190	0.105 to 0.275	< 0.0001

AA, arachidonic acid; CAD, coronary artery disease; DHA, docosahexaenoic acid; DHA/AA, ratio of DHA and AA; EPA, eicosapentaenoic acid; EPA/AA, ratio of EPA and AA; ROC, receiver operating characteristic.

## Discussion

In the present study, we have several novel findings. First, we found that AA may be associated with the increased risk of CAD in young Chinese patients after adjustment for multiple conventional risk factors. EPA may play a protective role on the risk of CAD, which was not observed for DHA. Second, the ratio of EPA/AA can increase the predictive value for diagnosing CAD than EPA or AA alone. Third, the level of EPA/AA in young Chinese individuals was very low, which may contribute to the high prevalence of premature CAD in China.

EPA can reduce the levels of atherogenic lipoproteins (e.g., triglycerides and remnant lipoprotein cholesterol), oxidative stress, and inflammatory cytokines. Furthermore, EPA can also improve endothelial function, inhibit foam cell formation, plaque progression and rupture, platelet aggregation, and thrombus formation (19). All of these factors contribute to the protective effect of EPA on the risk of CAD. In contrast, AA is a metabolic precursor for many prostaglandins, leukotrienes, thromboxanes, and other oxidized derivatives, and is considered to be a predominantly pro-inflammatory fatty acid (19). Therefore, the ratio of EPA/AA can be regarded as a balance of anti-inflammatory/pro-inflammatory and anti-aggregatory/pro-aggregatory status *in vivo*. Although both EPA and DHA were referred to as LC omega-3 PUFAs and significantly correlate with each other, they may have different biologic effects. The inhibition of cholesterol crystalline domains by EPA but not DHA, may result in a difference in endothelial function (20). Furthermore, EPA is more efficiently incorporated into HDL particles, which can increase its ability to inhibit HDL oxidation than DHA (21). Previous clinical studies also supported these basic research findings. Nishizaki et al. enrolled 1,119 patients from a metropolitan area in Japan, and found that individuals with the lowest tertiles of EPA/AA (≤ 0.33) had a greater probability of acute coronary syndrome (OR 3.14, 95% CI 1.16–8.49), while the similar association was not observed for DHA/AA (7). In contrast, they updated the sample size with 1,733 patients, and reported that a high DHA/AA ratio was significantly associated with a low risk of ACS among men (OR = 0.389; 95%CI 0.211–0.716), however, such association was not significant in women (10). A recently individual-participant data meta-analysis comprising 3,022 incident CHD cases (13,104 controls) showed that although circulating DHA was related to lower CAD risk in the fully adjusted model (OR 0.85; 95% CI, 0.76–0.95, per standard unit increment), there was significant heterogeneity among studies and the effect was modified by study design (22). In the current study, we found that DHA was not associated with the risk of CAD. Although the ratio of DHA/AA showed a reverse association with the odds ratio of CAD, this effect was mainly driven by the level of AA. The AUC for predicting CAD was similar in AA and DHA/AA, which further supports that detection of DHA cannot further provide additional information for determining the risk of CAD based on the level of AA.

Based on our results, we proposed that supplementing with EPA (to increase the ratio of EPA/AA), but not DHA, may play a role in the prevention of CVD. The Japan EPA Lipid Intervention Study (JELIS) showed that treating dyslipidemic patients with highly purified EPA and statins significantly reduced the incidence of major adverse cardiac events, compared with that observed in patients administered statins alone (23). Similarly, the REDUCE-IT (Reduction of Cardiovascular Events with Icosapent Ethyl-Intervention Trial) showed that among statin-treated patients with elevated triglycerides and CVD or diabetes, highly purified EPA (icosapent ethyl) can substantially reduce the burden of first, subsequent, and total ischemic events (24). In contrast, those studies used a combination of EPA and DHA did not result in a significant difference in the risk of cardiovascular events (25–27). Although not fully explored, the different effects of EPA and DHA on cardiovascular health may attribute to the inconsistent results. However, limited data had been conducted on the young Chinese population, which is urgently needed.

Another astonishing finding in the current study was that the level of EPA/AA was unexpectedly low in our study. As an Asian country, we previously presumed the ratio of EPA/AA may be similar to that reported in Japan. The Hisayama study from the Japanese general population showed that the median ratio of EPA/AA was 0.41 (interquartile range 0.29–0.59) (28). Another study reported that in White, Japanese, and Japanese American men aged 40–49 years, the ratios of EPA/AA were about 0.09, 0.39, and 0.12, respectively (29). In our study, the median ratios of EPA/DHA were 0.09, 0.08 and 0.11 in all participants, CAD patients and controls, respectively, which was very similar with the Whites, but significantly lower than that in Japanese. The westernization of food customs in China during the past decades may explain this phenomenon. Dietary habits directly influence the EPA/AA ratio. Meat is abundant in AA. The population-based China Health and Nutrition Survey, followed across 24 years, showed that there was a great transition from the traditional to the Western diet, especially on animal source foods (30). These data call an urgently needed to change the dietary profiles of PUFAs to prevent the epidemic of premature CVD in China.

Several limitations should be noted in the current study. First, the case-control design of the study can only show the association, but not causality among the detected free fatty acids and their ratios with CAD. Further prospective cohort studies should be performed to support the findings in our study. Second, other clinical subtypes of ischemic heart disease like coronary microvascular dysfunction may be neglected and not been excluded in the control group by the traditional criteria of CAD (≥ 50% stenosis of the lumen diameter in at least one major coronary artery). However, we considered that the inclusion of potential CAD with coronary microvascular dysfunction in the case group, may weaken, rather than increase the difference of interested markers between the case and control groups. Therefore, such limitation may not alter the predicting value of AA, EPA and the ratio of EPA/AA for CAD. Third, serum levels of fatty acids were closely related to dietary intake. We only included participants from Guangdong province, Southern China, therefore, the results cannot be extended to people from other regions. Fourth, some medicine may interact with the metabolism of fatty acids, e.g., non-steroidal anti-inflammatory drug, was not recorded in all the participants, which is an underlying confounding factors in the current study. Fifth, dietary intake of the interested fatty acids were not recorded in the current, which make it difficult to access whether the low level of EPA/AA ratio was caused by dietary pattern or genetic factors. However, we think this would not alter the effect of circulating EPA/AA ratio on predicting the risk of CAD in our study.

## Conclusion

EPA, but not DHA may play a protective role in CAD, while AA may be associated with the increased risk of CAD in young Chinese patients. The ratio of EPA/AA can increase the predictive value for diagnosing CAD than EPA or AA alone. Further studies are needed to explore the effects of EPA supplements for decreasing the risk of CAD in young Chinese individuals.

## Data availability statement

The raw data supporting the conclusions of this article will be made available by the authors, without undue reservation.

## Ethics statement

The studies involving human participants were reviewed and approved by the Ethics Committee of Shunde Hospital, Southern Medical University, China. The patients/participants provided their written informed consent to participate in this study.

## Author contributions

XL, LS, YunH, and YulH were responsible for the initial plan, study design, conducting the study, and data interpretation. XL, LS, WW, MQ, JL, WL, SH, and MH were responsible for data collection and data extraction. XL and YulH performed the statistical analysis and manuscript drafting. XL, LS, JW, YunH, and YulH were responsible for interpreting the data and critically revised the manuscript. JW, YunH, and YulH were guarantors and had full access to all of the data, including statistical reports and tables, and took full responsibility for the integrity of the data and the accuracy of the data analysis. All authors contributed to the article and approved the submitted version.
